# Influence of Plastic Strain Accumulation in Continuous Ingots during ECAP on Structure and Recrystallization Temperature of AlCu4MgSi Alloy

**DOI:** 10.3390/ma13030576

**Published:** 2020-01-25

**Authors:** Paweł M. Nuckowski, Przemysław Snopiński, Tomasz Wróbel

**Affiliations:** 1 Department of Engineering Materials and Biomaterials, Silesian University of Technology, 18A Konarskiego Street, 44-100 Gliwice, Poland; przemyslaw.snopinski@polsl.pl; 2 Department of Foundry Engineering, Silesian University of Technology, 7 Towarowa Street, 44-100 Gliwice, Poland; tomasz.wrobel@polsl.pl

**Keywords:** aluminum alloy, continuous ingot, severe plastic deformation (SPD), equal channel angular pressing ECAP, microstructure, high-temperature X-ray diffraction (HT-XRD)

## Abstract

This work attempts to process AlCu4MgSi (AW 2017A) alloy continuous ingots by means of the equal channel angular pressing (ECAP) method. The equal channel angular pressing (ECAP) technique has been widely investigated in recent years as the most promising severe plastic deformation (SPD) method. The presented research was focused on the precise determination of the phase composition of the precipitates formed in the AlCu4MgSi alloy and its influence on intensive plastic deformation. In the second stage, the research was focused on explaining the recrystallization process. With the use of the high-temperature X-ray diffraction (HT-XRD) technique, the changes in the dislocation substructure at various recrystallization annealing temperatures were analyzed.

## 1. Introduction

Among the currently used engineering materials, Al-Cu alloys, classified under the 2xxx series, have wide-ranging industrial applications. Due to their very good strength-to-weight ratio, they are used for the development of higher-performance parts and constructions for aircraft and the automotive industry [1,2,3]. The Cu addition in these alloys makes them prone to precipitation hardening through the formation of fine-dispersive precipitates of the intermetallic phase Al_2_Cu, which causes a significant increase of the strength properties. Aluminum alloys from the 2xxx series can be cold plastically treated, between the supersaturation and natural aging (state T3), which leads to an increase in the strength through an increase in the dislocation density as well as a uniform distribution of the secondary phase particles (secondary particle precipitation) in the solid solution α-Al. In order to improve the plasticity, full annealing is applied (state 0), consisting of annealing at a temperature within the scope of 400 to 425 °C, followed by slow cooling together with the furnace to a temperature below 230 °C, from which the alloy is treated plastically. In such a variant, the supersaturation and artificial aging occur after the deformation. At present, the most commonly used cold plastic treatment method for 2xxx alloys is die forging. In turn, hot plastic processing is performed by way of extrusion, which makes it possible to produce high-strength profiles [3,4,5,6]. However, this technology has a few weak points, as demonstrated by Schikorra et al. [7]. The main flaw is disadvantageous grain growth as a result of dynamic and static recrystallization, leading to a drop of the strength properties. The use of plastic treatment methods based on intensive plastic deformation (SPD—severe plastic deformation) [8,9,10,11,12] opens up new possibilities in the plastic processing of well-recognized aluminum alloys from the 2xxx series. A combination of the undoubted benefits of the SPD methods, i.e., the possibility of grain refinement and energy increase in the form of a developed dislocation substructure (subgrains), with the possibilities of complex thermal treatment of multi-phase alloys constitutes a solid basis for the development of innovative methods of producing light metal materials with increased strength properties. The investigations performed by Lechner et al. [13] on Al-Cu-Mg alloys deformed with the use of high-pressure torsion (HPT) point to a beneficial combination of precipitation hardening with the grain refinement mechanisms induced by intensive deformation. In turn, Murayama et al. [14] demonstrates a significant effect of deformation energy accumulation on the kinetics of phase θ in the supersaturated solution Al-Cu. An interesting approach to the subject was demonstrated by Namdar et al. [15], who compared the mechanical properties and the fatigue strength of the commercial aluminum alloy AW 2011 in four different variants of thermal and plastic treatment with the use of the equal channel angular pressing method (ECAP). They demonstrated that aluminum bars processed by the ECAP method between supersaturation and artificial aging characterize in a yield point that is higher by 230 MPa as well as a 50% increase of fatigue strength, compared to bars processed with the conventional techniques in state T6, i.e., after supersaturation and artificial aging. In addition, the study [16] describes plastic treatment of the Al-3%Mg alloy by means of the ECAP method applied after precipitation hardening (state T8) as well as between supersaturation and artificial aging (state T9). It was proved that the significant improvement of the mechanical properties was a consequence of grain refinement, solid solution reinforcement, and dislocation accumulation (formation of a developed substructure).

## 2. Materials and Methods 

The presented work analyzes the possibility of plastic deformation performed by the equal channel angular pressing method of an AlCu4MgSi alloy ingot (AW 2017A) produced in the process of horizontal continuous casting. The plastic treatment was combined with thermal treatment. The performed research was divided into two stages. In the first stage, the ECAP plastic treatment of the ingot was carried out between the procedure of supersaturation and natural aging (state T3) by way of an analysis of the effect of severe plastic deformation on the structure. The obtained results were compared with the results for the ingot in state T4 (supersaturation and natural aging). The second stage focused on the explication of the recrystallization process. With the application of the high-temperature X-ray diffraction technique (HT-XRD), the changes in the dislocation substructure were analyzed in three variants of the ingots thermal treatment in state T3 (supersaturation + ECAP + natural aging), consisting in recrystallizing annealing performed at temperatures T: 260, 390, and 510 °C.

**Figure 1:**
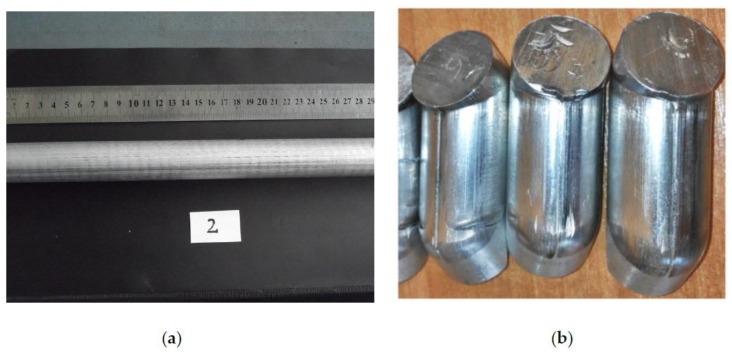
AlCu4MgSi alloy (AW 2017A), (**a**) ingot obtained in horizontal continuous casting, (**b**) ingot fragments plastically treated by equal channel angular pressing (ECAP).

A continuous ingot of AlCu4MgSi alloy that was 30 mm in diameter (Figure 1a) was made with the use of a test bench for horizontal continuous casting being at the disposal of the technological laboratory of Silesian University of Technology’s Department of Foundry Engineering. The casting was carried out through sequential removal of the ingot at the mean casting rate of 40 mm/min. The temperature of the water cooling the crystallizer was within the scope of 45 to 55 °C, with the flow of 0.5–1.2 L/min in a closed cycle. The detailed parameters of the applied process of horizontal continuous casting were presented in an earlier study of the authors [17]. 

The chemical composition of the obtained ingot was determined with the use of a GD OES spectrometer (glow discharge optical mission spectroscopy) LECO GDS500A and compiled with the current standard ISO and EN (Table 1). Next, the cut-down fragments of the ingot underwent thermal treatment consisting in supersaturation (ST) at 525 °C for 3 h, followed by cooling in water.

The process of pressing through the angular channel (ECAP) was performed at room temperature with the use of a hydraulic press with the force of 1600 kN. The AlCu4MgSi alloy ingots, after supersaturation, were processed by means of an ECAP 120° die in a single pass at a constant deformation rate of 2 mm/s, which corresponded to the equivalent strain of approximately 0.6. Next, the plastically processed material (Figure 1b) underwent natural aging.

In the first stage, the effect of severe plastic deformation as well as thermal treatment on the structure of the AlCu4MgSi alloy ingot was examined. The microstructure observations on the cross-sections (TD) of the ingots in states T3 and T4 were made under a light microscope ZEISS AxioObserver (Jena, Germany) with the use of the polarized light technique. The analysis of the structure and the chemical composition was performed on a scanning microscope PhenomProX (Phenom-World Eindhoven, Noord-Brabant, the Netherlands) equipped with an energy-dispersive X-ray spectrometer (EDS). In order to determine the phase composition of the AlCu4MgSi alloy, X-ray diffraction tests were carried out. To that end, an X-ray diffractometer X’Pert Pro MPD by Panalytical (Almelo, the Netherlands) was used, which was equipped with a copper anode lamp (λKα = 0.154 nm) (Panalytical, Almelo, the Netherlands) as well as a PIXcel 3D detector (Panalytical, Almelo, the Netherlands) on the diffracted beam axis. The diffraction lines were recorded in the Bragg–Brentano geometry in the angular scope of 15–90° [2*θ*], with the step of 0.03° and the step time of 80 s. The samples of the ingots and the material after the thermal and plastic treatment ECAP were cut out parallel to the ED direction. The analysis of the obtained diffraction patterns was made in the Panalytical High Score Plus software (Version3.0e), containing a dedicated flat file base of PAN-ICSD phase identification. 

**Figure 2:**
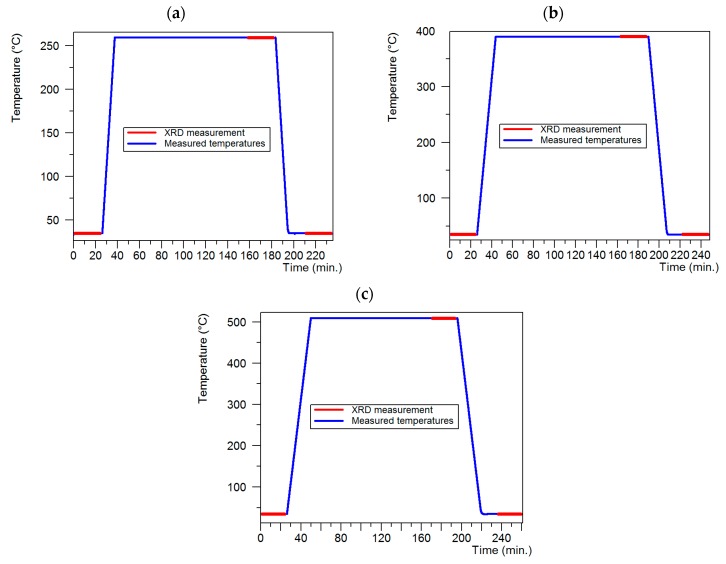
Diagrams showing the thermal treatment course in the Anton Paar HTK16 chamber with the marked areas of the X-ray diffraction measurement (red line); (**a**) 0.4Tt (260 °C), (**b**) 0.6Tt (390 °C) and (**c**) 0.8Tt (510 °C).

The second stage focused on the analysis of the effect of the thermal treatment temperature on the recrystallization degree of the deformed alloy AlCu4MgSi (T3). For that purpose, the high-temperature X-ray diffraction technique (HT-XRD) was applied. The measurements were made in a Panalytical diffractometer equipped with a high-temperature chamber Anton Paar HTK 16 (Graz, Austria) with a platinum heating unit. Three identical samples were collected from the thermally treated and ECAP deformed ingot in state T3. For each sample, a different annealing temperature in the Anton Paar HTK 16 chamber was applied, which was selected on the basis of the melting point of phase αAl (αAl Tt ≈ 640 °C) and equal to 0.4Tt (260 °C), 0.6Tt (390 °C), and 0.8Tt (510 °C). The samples were heated and cooled at the rate of 20 °C/min. The diffraction measurements for each sample were made in three stages: before the annealing at 35 °C, at the maximal annealing temperature after 2 h of holding, and after cooling at 35 °C. The diagrams of the thermal treatment conditions in the Anton Paar chamber for each sample have been given in Figure 2. 

The average crystallite size as well as the lattice microstrain were calculated based on the four diffraction lines recorded for phase fcc-αAl (111), (200), (220) and (311), with the use of Equation (1) of Williamson Hall [18,19].

(1) $$Bcos\theta =\left[\frac{K\lambda }{D_{v}}\right]+\left[4\varepsilon sin\theta \right]$$

where *Dv* is the average crystallite size, *λ* is the X-ray wavelength, *K* is Scherrer’s constant depending on the type of crystal lattice, *ϴ* is the Bragg angle, B is the line broadening, and ε is the root mean square of the microstrain. The dislocation density was estimated according to the Rietveld method [20] from following Equations (2)–(4):

(2) $$\rho ={\left(\rho_{D}\cdot \rho_{s}\right)}^{1/2}$$

(3) $$\rho_{D}=3/D_{v}^{2}$$

(4) $$\rho_{s}=\varepsilon^{2}/b^{2}$$

where *ρD* is the dislocation density due to domains, *ρs* is the dislocation density due to the microstructure, and *b* is Burger’s vector.

## 3. Results and Discussion

### 3.1. Metallographic Microscopic Studies 

**Figure 3:**
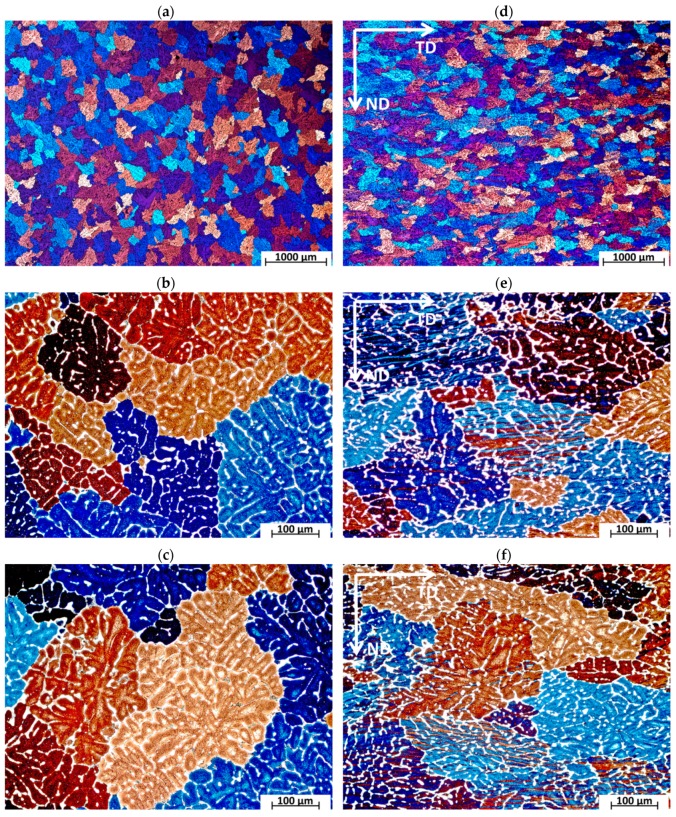
Microstructure of the AlCu4MgSi alloy ingot; (**a**–**c**) after annealing at 525 °C/3 h and natural aging (state T4); (**d**–**f**) after supersaturation + ECAP deformation and natural aging (state T3).

**Figure 4:**
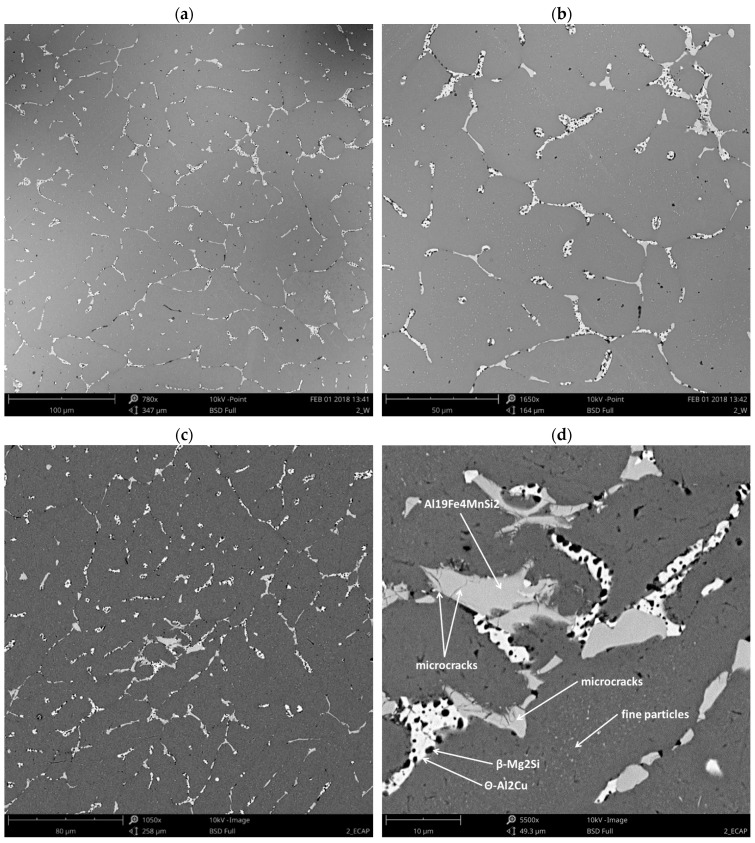
Microstructure of the AlCu4MgSi alloy ingot; (**a**,**b**) after annealing at 525 °C/3 h and natural aging (state T4); and (**c**,**d**) after supersaturation + ECAP deformation and natural aging (state T3).

**Figure 5:**
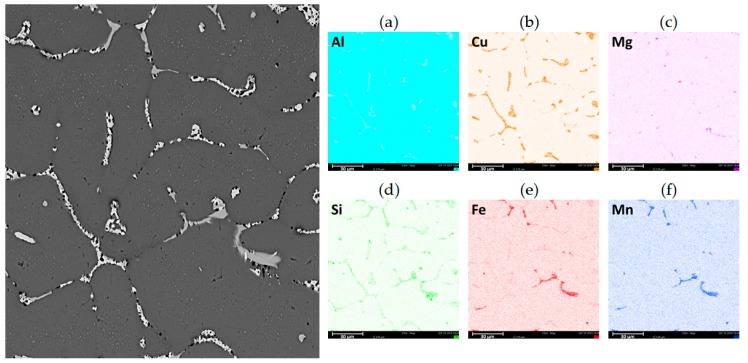
Microstructure of the AlCu4MgSi alloy ingot after annealing at 525 °C/3 h and natural aging (state T4) and results of chemical composition analysis in this area, map for (**a**) Al, (**b**) Cu, (**c**) Mg, (**d**) Si, (**e**) Fe, and (**f**) Mn.

**Figure 6:**
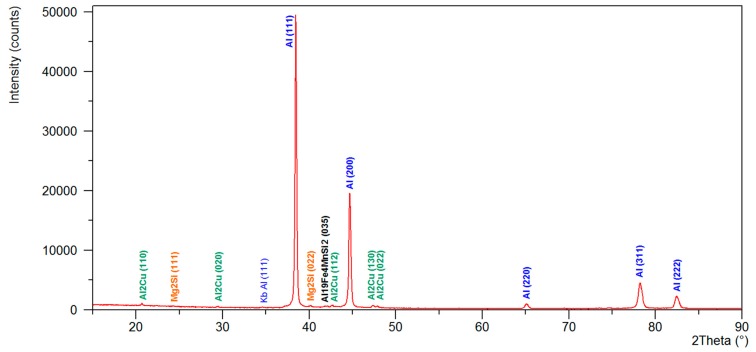
X-ray diffraction patterns of AlCu4MgSi alloy ingots after annealing at 525 °C/3 h and natural aging (state T4).

The metallographic observations (Figure 3a–c) of the AlCu4MgSi ingot after supersaturation at 525 °C/3 h as well as natural aging (T4) showed the presence of mainly uniaxial grains of the solid solution α-Al with irregular shapes and diversified sizes in the structure. The averaged value of the surface area per grain calculated with the Jeffries method equals approximately 0.06 mm^2^. The grains characterize in a diversified crystallographic orientation, which is revealed in a change of their color and contrast during the observations in polarized light. It can be observed that the solid solution α-Al characterizes in a typical dendritic structure with a characteristic network of non-equilibrium phase precipitations, which are localized in the interdendritic areas. The tests performed with the use of a scanning electron microscope (Figure 4a–b) made it possible to evaluate the morphology of these precipitations. The observed precipitates assume the form of a complex network as well as numerous isolated globular forms being a remainder of the incomplete dissolution of the non-equilibrium phases during the supersaturation process. The chemical composition analysis in micro-areas (Figure 5a–f and Table 2) showed that those are mainly intermetallic phases θ-Al_2_Cu with a tetragonal lattice I4/mcm (white precipitation areas in Figure 4a–d) and β-Mg_2_Si with a regular lattice Fm-3m (dark points situated in the white θ-Al_2_Cu precipitation areas in Figure 4a–d), which was also confirmed by the qualitative X-ray phase analysis (Figure 6). The analysis of the contrast in the precipitation areas as well as the results of the X-ray microanalysis point to a minor share of a complex multi-component phase rich with iron and manganese (gray precipitation areas). Its identification as phase Al_19_Fe_4_MnSi_2_ with a regular lattice Im-3 was also possible owing to the use of the X-ray diffraction analysis (XRD). Within the precipitation lattice, in the interdendritic areas, uniformly oriented, fine-dispersive crystals of phase Al_2_Cu are present, which were formed as a result of secondary crystallization from the solid solution in the process of natural aging.

**Figure 7:**
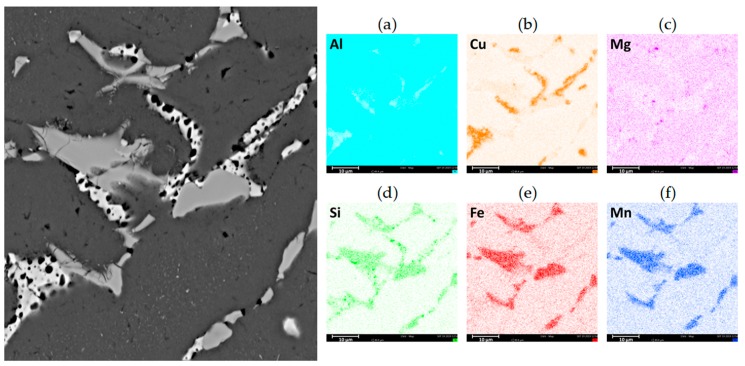
Microstructure of the AlCu4MgSi alloy ingot after supersaturation + ECAP deformation and natural aging (state T3) and results of chemical composition analysis in this area, map for (**a**) Al, (**b**) Cu, (**c**) Mg, (**d**) Si, (**e**) Fe, and (**f**) Mn.

The microstructure of the AlCu4MgSi ingot after one ECAP pressing cycle as well as the thermal treatment (state T3) has been shown in Figure 3d–f. We can see a change in the grains’ geometry in the form of their elongation in the transverse direction (TD) with respect to the structure of an ingot without plastic treatment (state T4). This happens because in the ECAP process, the material deformation is realized by way of pure shear and depending on the applied deformation path, the microstructural changes are different in character. In the case of deformation by means of Path A (one ECAP cycle), the shearing occurs in two perpendicular planes in such a way that the grains become elongated in the transverse direction (TD) during the observation of the cross-section of the sample [21]. This is confirmed by the microscopic examinations performed at higher magnifications, where on the cross-sections, it is possible to observe numerous deformation bands localized within the grains. These bands, which are easy to notice owing to the use of polarization contrast, are oriented parallel to the TD direction and do not cross the grain boundaries. A similar polarization contrast within the neighboring grains points to the interaction of the bands in one slide system. The observations made under a SEM microscope (Figure 4c–d) confirmed that the non-equilibrium phase precipitation lattice maintained a structure close to the initial one (before deformation). In the microphotographs (Figure 4d and Figure 7), characteristic microcracks were observed, which were located mainly in the precipitation areas of phase Al_19_Fe_4_MnSi_2_, which was confirmed by the surface analysis EDS (Figure 7a–f and Table 3). The formation of a similar phase Al(FeMn)Si in 6xxx aluminum alloys with a similar content of Fe and Mn was described in the study [22]. In addition, Balitchev et al. [23], while performing thermodynamic modeling of the Al-Fe-Mn-Si system, describe the AlFeSi and AlMnSi phases that are often present in this system. They also point to the possibility of the formation of a similarly structured phase Al_16_Fe_1.7_Mn_2.3_Si_3_. In their research, G. Irizalp et al. [24] demonstrate that in cast aluminum alloys, manganese reacts with iron, forming multi-component intermetallic phases in the form of precipitations, which assume different forms and chemical compositions, depending on the amount of manganese in the alloy. The phases present in the Al-Fe-Mn-Si system characterize in high hardness as well as thermal stability in the scope of the supersaturation temperatures of aluminum alloys, which can constitute an obstacle in the process of plastic treatment. The microcracks within the precipitations of the described phase Al_19_Fe_4_MnSi_2_ observed in the analyzed material can be a confirmation of this thesis. In turn, the results presented in the work [25] suggest also a strict connection between the strength properties of aluminum alloys and the morphology of the hard phases from the Al-Fe-Mn-Si system. A significant reduction of impact strength was recorded with the precipitates with the shape of needles/plates observed in the microstructure, which created complex forms, the so-called Chinese scripts. More advantageous with regard to the mechanical properties was proved to be the compact multi-wall shape of the precipitations of the Fe and Mn containing phases. The effect of this type of microdefect in the Al_19_Fe_4_MnSi_2_ phase areas on the mechanical properties, especially the impact strength, can constitute the basis for further research in this field.

### 3.2. High Temperature X-ray Diffraction Studies 

**Figure 8:**
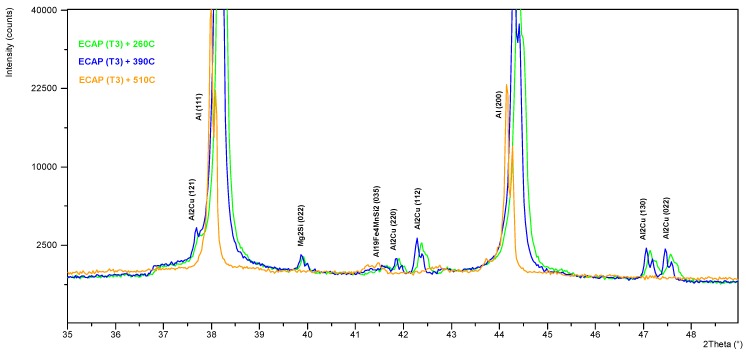
High-temperature X-ray diffraction analysis of AlCu4MgSi alloy after the ECAP process (T3), measurement conducted at temperatures 260 °C (green line), 390 °C (blue line), and 510 °C (orange line).

**Figure 9:**
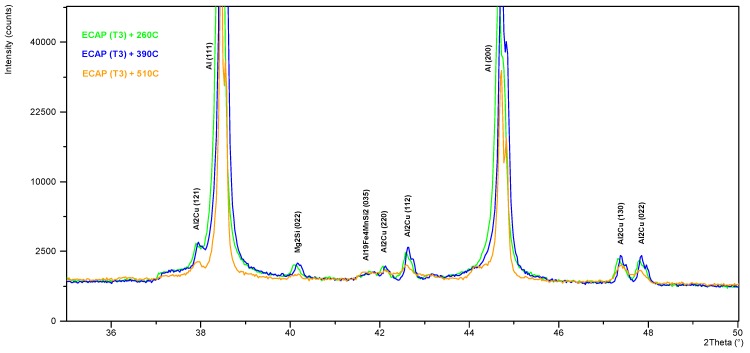
X-ray diffraction patterns of AlCu4MgSi alloy samples after the ECAP process (T3) recorded at 35 °C after cooling from the annealing temperatures 260 °C, 390 °C, and 510 °C.

**Figure 10:**
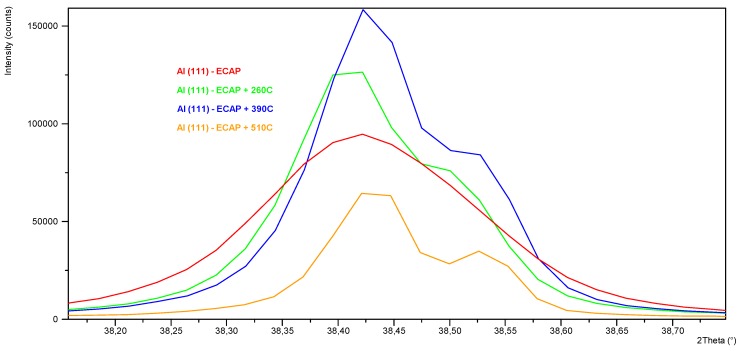
Profile of α-Al (111) diffraction line after the ECAP process (T3) and after annealing at 260 °C, 390 °C, and 510 °C as well as cooling.

The use of a high-temperature chamber HTK 16 Anton Paar enabled precise control of the course of the heating and cooling curves, with a simultaneous measurement of the phase composition in the selected points of the process. The diffraction patterns obtained for the samples after one ECAP cycle in state T3 annealed at 260, 390, and 510 °C, with the use of the HTK 16 chamber, have been shown in Figure 8 and Figure 9. The compilation of the diffraction spectra obtained by means of the HT-XRD technique (Figure 8) points to the presence of θ-Al_2_Cu, β-Mg_2_Si, and Al_19_Fe_4_MnSi_2_ phase precipitations in the solid solution α-Al, at the temperatures 260 and 390 °C. On the diffraction pattern obtained at 510 °C, lines coming from the solid solution and the Al_19_Fe_4_MnSi_2_ phase were observed, while no characteristic diffraction lines coming from the θ-Al_2_Cu and β-Mg_2_Si phases were recorded. So, it can be stated that the described phase of the Al-Fe-Mn-Si system is thermally stable in the scope of the applied thermal treatment temperatures, whereas phases θ and β dissolve in the solid solution at 510 °C. This effect is disadvantageous due to the complete dissolution of the fine-dispersive precipitates, mainly of phase θ, which reinforce the solid solution and hinder the grain growth during recrystallization [26]. With the applied cooling rate (20 °C/min), these phases can precipitate in the interdendritic areas, causing a drop of impact strength. With the temperature increase, a slight shift of the diffraction lines toward the lower angle values 2*θ* is observed, which is caused by the effect of thermal expansion. The qualitative X-ray phase analysis (Figure 9) performed right after the samples had been cooled from the annealing temperature to 35°C at the rate of 20 °C/min showed the repeated precipitation of phases θ and β from the supersaturated solid solution α-Al. It can be inferred from the analysis of the diffraction spectrum profile of the line from the planes (111) of solution α-Al that the lines recorded for the sample in state T3 (supersaturation + ECAP + natural aging) characterize in the biggest broadening. With the increase of recrystallization temperature, a division into components Kα1 and Kα2 of the X-ray is observed (Figure 10). This effect can be related to the process of restructuring and annihilation of the linear defects. As is demonstrated by Chuan-hai Jiang et al. [27], a typical diffraction spectrum of plastically deformed aluminum is close to the Gauss distribution, which is caused by the agglomeration of linear defects as well as deformation of the crystal lattice, consisting in its bend or twist in the area of the domains. Line (111) α-Al recorded for the sample annealed at 510 °C for 2 h exhibits a characteristic profile of a recrystallized structure. 

In order to determine the broadening of the diffraction lines recorded from the planes (111), (200), (220), and (311) α-Al, the components K_α1_ and K_α2_ were separated by means of the Rachinger method. The mean size of the crystallites as well as the dislocation density due to the size effect (*ρ_D_*) and the dislocation density due to lattice microstrain (*ρ_S_*), which was calculated for the analyzed thermal treatment variants, have been presented in Table 4. The sample after plastic treatment in state T3 characterizes in the average crystallite size (35 nm) and the average dislocation density 5.30 × 10^14^ m^–2^. The development of the substructure and the increase of the dislocation density in aluminum processed with the use of severe plastic deformation depends on the stress, which has been discussed in the study [28]. In addition, the agglomeration of point defects in the supersaturated solution intensifies the process of subgrain formation during the plastic treatment [29,30]. The significant difference between the dislocation density due to the size effect (2.36 × 10^15^ m^−2^) and the dislocation density due to lattice microstrain (1.19 × 10^13^ m^−2^) is connected with the energy balance. As it was demonstrated in the works [28,31], a comparison of the energy of the dislocation groups uniformly distributed with respect to the volume with the energy of the dislocation in the walls of the dislocation cells clearly shows that with a specific dislocation energy, it is more advantageous energy-wise to create cells instead of maintaining a uniform distribution. This relation, regardless of the applied treatment temperature, is proven for each of the analyzed samples. The mean size of the crystallite in the sample annealed at 260 °C/2 h increased to 67 nm, while the mean dislocation density decreased by an order of magnitude (5.74 × 10^13^ m^−2^), with the maintained difference of two orders of magnitude between *ρ_D_* = 6.57 × 10^14^ m^−2^ and *ρ_S_* = 5.00 × 10^12^ m^−2^. While analyzing the recorded full profile of line (111) α-Al, one can state that with annealing at 260 °C, the observed change in the size of the domains and the dislocation density is caused by the thermally activated recovery process, consisting of the annihilation of a part of the dislocations and regrouping of the remaining ones (the so-called polygonization). Annealing at 390 °C/2 h caused a decrease of mean dislocation density to 1.81 × 10^13^ m^−2^, with the crystallite size of the order to 80 nm, and the analysis of the diffraction lines profile shows that the recrystallization threshold has been crossed. An increase of the difference between *ρ_D_* (4.66 × 10^14^ m^−2^) and *ρ_S_* (7.04 × 10^11^ m^−2^) can point to small grains with a high dislocation concentration or large grains with low dislocation density being present in the microstructure. A similar relation has been described in [32]. For the sample annealed at 510 °C/2 h, the recorded mean crystallite size was estimated to be 94 nm, whereas the dislocation density—1.80 × 10^13^ m^−2^, which is the same value as in the case of the sample annealed at 390 °C. Based on the obtained results, we can state that at 510 °C, beside the above-mentioned processes, grain growth takes place as a result of secondary recrystallization. As it was demonstrated before, this temperature variant is also disadvantageous due to the dissolution of phases Al_2_Cu and Mg_2_Si in the solid solution α-Al, which significantly reduces the fine-dispersive crystallites of phase θ, which were obtained as a result of precipitation hardening. 

## 4. Conclusions

The research performed on the plastic treatment of an AlCu4MgSi alloy ingot with the use of the ECAP method and by means of high temperature X-ray diffraction (HT-XRD) for the analysis of the recrystallization process made it possible to draw the following conclusions.

It is possible to obtain severe plastic deformation of a continuous ingot made of AlCu4MgSi (AW 2017A) alloy with the use of the ECAP method, which is performed between the procedure of supersaturation and natural aging. This process leads to the changes in the microstructure characteristic to this type of plastic treatment in the form of agglomeration of deformation bands.

The presence of iron and manganese in the analyzed alloy leads to the formation of Al_19_Fe_4_MnSi_2_ phase precipitations with a compact multi-wall shape. Severe plastic deformation of the supersaturated alloy AlCu4MgSi in one pressing cycle of ECAP causes the presence of numerous microcracks in the precipitation areas of this phase. 

Annealing at 260 °C/2 h leads to an increase of the mean size of the crystallites from 35 nm (state T3 before annealing) to 67 nm. The observed change in the domain size and the dislocation density (from 5.30 × 10^14^ to 5.74 × 10^13^ m^−2^) is mainly caused by the thermally activated recovery process. It was demonstrated that annealing at 390 °C/2 h causes a decrease of the mean dislocation density to 1.81 × 10^13^ m^−2^, with the crystallite size of the order of 80 nm. In turn, the analysis of the diffraction lines profile shows that the recrystallization threshold was crossed. The third applied temperature variant (510 °C/2 h) is disadvantageous due to the dissolution of phases Al_2_Cu and Mg_2_Si in the solid solution α-Al; this significantly reduces the fine-dispersive crystallites of phase θ, which was obtained as a result of precipitation hardening. 

High-temperature X-ray diffraction (HT-XRD) is an effective tool for the analysis of the phase transformations in the function of temperature. It was proved that this method provides broad analytical possibilities in the planning of the thermal treatment process, especially of complex multi-phase aluminum alloys, in which, as a result of severe plastic deformation, the accumulated energy causes a shift of the recrystallization threshold.

## Figures and Tables

**Table 1:** Chemical composition of studied AlCu4MgSi (EN AW-2017A) alloy.

Material Designation		Elements Concentration [% wt.]										
ISO:	EN:	Element	Si	Fe	Cu	Mn	Mg	Cr	Zn	other	Zr+Ti	Al
AlCu4MgSi	AW-2017A	min.	0.2	≤0.7	3.5	0.4	0.4	≤0.1	≤0.25	≤0.15	≤0.25	balance
		max.	0.8	-	4.5	1.0	1.0	-	-	-	-	-
Studied ingot (GD OES)			0.52	0.28	4.45	0.82	1.00	0.05	0.22	0.03	0.01	balance

**Table 2:** Results of chemical composition analysis for area shown in Figure 5.

Element Symbol	Atomic Concentration [%]	Weight Concentration [%]
Al	96.12	93.73
Cu	1.64	3.76
Mg	1.31	1.15
Si	0.50	0.51
Fe	0.11	0.23
Mn	0.31	0.62

**Table 3:** Results of chemical composition analysis for area shown in Figure 7.

Element Symbol	Atomic Concentration [%]	Weight Concentration [%]
Al	95.93	91.60
Cu	2.44	5.50
Mg	0.16	0.14
Si	0.11	0.11
Fe	0.69	1.35
Mn	0.67	1.31

**Table 4:** Structural parameters of the AlCu4MgSi aluminum alloy determined by X-ray diffraction peak analysis. *D_v_* = domain size; *ρ_D_* = dislocation density due to size effect; *ρ_S_* = dislocation density due to lattice microstrain; *ρ* = total dislocation density.

Condition	*D_v_* (nm)	*ρ_D_* (m^−2^)	*ρ_S_* (m^−2^)	*ρ* (m^−2^)
ECAP (T3)	35	2.36 × 10^15^	1.19 × 10^13^	5.30 × 10^14^
ECAP (T3) + 260 °C	67	6.57 × 10^14^	5.00 × 10^12^	5.74 × 10^13^
ECAP (T3) + 390 °C	80	4.66 × 10^14^	7.04 × 10^11^	1.81 × 10^13^
ECAP (T3) + 510 °C	94	3.37 × 10^14^	9.58 × 10^11^	1.80 × 10^13^
